# Application of Copy Number Variation Detection to Fetal Diagnosis of Echogenic Intracardiac Focus During Pregnancy

**DOI:** 10.3389/fgene.2021.626044

**Published:** 2021-03-26

**Authors:** Yaxian Song, Jingjing Xu, Hongmiao Li, Jiong Gao, Limin Wu, Guoping He, Wen Liu, Yue Hu, Yaqin Peng, Fang Yang, Xiaohua Jiang, Jing Wang

**Affiliations:** ^1^Department of Obstetrics and Gynecology, The First Affiliated Hospital of USTC, Division of Life Sciences and Medicine, University of Science and Technology of China, Hefei, China; ^2^Clinical Laboratory of Beijing Genomics Institute (BGI) Health, BGI-Shenzhen, Shenzhen, China; ^3^Department of Obstetrics and Gynecology, The First Affiliated Hospital of Anhui University of Science and Technology, Anhui University of Science and Technology, Huainan, China

**Keywords:** echogenic intracardiac focus, ultrasound soft markers, copy number variation, karyotype, congenital heart defects

## Abstract

Echogenic intracardiac focus (EIF) is one of the most common ultrasound soft markers (USMs) in prenatal screening. However, the association of EIF with chromosomal abnormalities is still controversial. From January 2018 to April 2020, a total of 571 fetuses with USMs in our center were enrolled, among which 150 (26.27%) presented EIFs. We analyzed the karyotype anomalies and copy number variations (CNVs) in fetuses who presented EIFs by comparing their ultrasound indications, maternal ages and gestational stages. There were no statistically significant differences in the incidence of chromosomal abnormalities between fetuses with EIFs and the fetuses with USMs (4.00 vs. 7.71%, *p* = 0.112). Additionally, the incidence of chromosomal abnormalities was not related to maternal age (4.10% in maternal age below 35 yeas vs. 3.57% in maternal age above 35, *p* = 1.000). Interestingly, after 28 weeks of gestation, fetuses with EIFs showed more chromosomal abnormalities (20.00%) than that in the group before 28 weeks of gestation (2.22%, *p* = 0.014), and this result was attributed to the detection of pathogenic CNVs. After birth, 25 of children conducted cardiac development re-examination. Among them, 9 (36%, 9/25) were diagnosed with congenital heart disease, primarily patent foramen oval and ventricular septal defects (7/9, 77.77%). We concluded that the appearance of EIFs in early or mid-trimester would not indicate an increased risk of fetal chromosomal abnormalities. However, the persistence of EIFs in late trimester was associated with a higher risk of pathology-related CNVs and its persistent appearance may indicate heart development defects after birth. Thus, our results suggest that CNV detection has its advantages in prenatal diagnosis, especially for those with EIFs that persist in the third trimester.

## Introduction

Ultrasound screening in the first and second trimesters of pregnancy is one of the most commonly performed genetic screenings. Besides sonographic structural defects, a group of findings classified as ultrasound soft markers (USMs) are often considered to indicate increased risk of underlying fetal aneuploidy, including echogenic intracardiac focus (EIF), thickening of the nuchal translucence, nasal bone dysplasia, echogenic bowel, single umbilical artery, short long bones, enlarged cisterna magna, cerebral ventriculomegaly, choroid plexus cyst, external left superior cavity, permanent right umbilical vein, right aortic arch, mild pyelectasis, and other conditions (Van Den Hof et al., 2005; Rembouskos et al., 2012; Choi et al., 2016; Lide et al., 2016).

EIF is defined as foci of echogenicity comparable to bone in the region of papillary muscle in either or both ventricles of fetal heart (Van Den Hof et al., 2005). EIF is one of the most common USMs in prenatal screening, with a prevalence ranging from less than 1% to 20% in different populations (Sepulveda and Romero, 1998; Wax et al., 2003). However, the association of EIF and aneuploidy is still controversial. For example, an increased incidence of trisomy 21 was found in fetuses who presented EIFs in high-risk pregnancies, however, some studies had failed to show this association (Dildy et al., 1996; Simpson et al., 1996; Achiron et al., 1997; Bromley et al., 1998; Manning et al., 1998; Winter et al., 2000). Additionally, previous results were mostly obtained based on traditional karyotyping, and their studies were always focused on trisomy syndromes, such as trisomy 21 syndrome. Recently, due to the availability of high-throughput sequencing, by measuring chromosomal microdeletions or microrepetitions, CNV-seq increases the detection efficiency of chromosomal abnormalities (Cohen et al., 2015; Committee on Genetics and the Society for Maternal-Fetal Medicine, 2016; Society for Maternal-Fetal Medicine et al., 2016; Clinical Genetics Group Of Medical Genetics Branch Chinese Medical Association et al., 2019). It was reported that 6.0% of fetuses presenting structural anomalies under ultrasound scanning had abnormal CNVs even karyotypes were normal (Wapner et al., 2012). Nevertheless, the CNV character in fetuses with EIFs is still seldom revealed.

It is generally accepted that most echogenic foci disappear with the progress of pregnancy and their constant presence may not imply poor pregnancy outcome (Simpson et al., 1996; Wolman et al., 2000; Wax et al., 2003; Chiu et al., 2019). Conflicting conclusions were elicited by the finding that euploid fetuses with EIFs showed cardiac diastolic dysfunction in the second trimester (Degani et al., 2001). Moreover, it was reported that the infants who showed fetal EIFs suffered more cardiac defects after birth than the general population (Goncalves et al., 2006).

Thus, in clinical consultation, although treatment of indications such as high risk in serological screening or in NIPT (Non-invasive Prenatal Testing) and fetal structural abnormalities is clear; perplexity arises when only EIF or USMs appear. In this investigation, to better estimate the risk of chromosomal abnormalities in fetuses with EIFs, we applied CNV-seq at a resolution of 100 kb simultaneously with conventional karyotyping in our prenatal diagnosis procedure and described the CNVs and karyotype abnormalities of fetuses with USMs and EIFs. The pregnancy outcomes from birth to 2 years of age were recorded and used to evaluate the association of EIFs with the presence of birth defects.

## Materials and Methods

### Editorial Policies and Ethical Considerations

The study was conducted with the Ethics Committee of the First Affiliated Hospital of University of Science and Technology of China (USTC). All procedures were performed in accordance with the ethical standards set forth in the Helsinki Declaration of 1964 and its latest amendments or comparable ethical standards. The participating pregnant women signed informed consent forms and agreed to allow the sequencing data to be used in research after anonymization.

### Patients and Study Design

This was a retrospective study. From January 2018 to April 2020, fetuses with USMs on prenatal diagnosis at the Prenatal Diagnosis Center of the First Affiliated Hospital of USTC were examined. Among them, fetuses who showed EIF were further investigated.

All of the pregnant women involved in this study received genetic counseling and provided written informed consent followed by amniocentesis or umbilical cord blood puncture. Using these samples, karyotype analysis and CNV-seq were conducted to identify fetal chromosomal abnormalities.

Ultrasound soft markers are based on sonographic findings, including EIF, thickening of the nuchal translucence, nasal bone dysplasia, echogenic bowel, single umbilical artery, short long bones, enlarged cisterna magna, hydrocystoma of neck, cerebral ventriculomegaly, choroid plexus cyst, external left superior cavity, permanent right umbilical vein, right aortic arch, mild pyelectasis, and other conditions.

Patients were excluded from this study when (1) the mother had previously delivered a child with a congenital defect; (2) the fetus displayed sonographic structural anomalies; (3) the couple had known genetic defects; or (4) the pregnancy was indicated to be high-risk based on serum screening or NIPT.

### Karyotype Analysis

Fetal amniotic fluid cells were cultured in complete medium (Baorong, Hangzhou, China). Cordocentesis samples were cultured in Cell Preservation Medium (Sinochrome, Shanghai, China). Karyotype analysis was performed on G-band metaphases prepared from amniotic fluid or cord blood samples with resolution between 320 and 420 bands. At least thirty metaphases were counted, and five from each sample were analyzed. Karyotypes are described according to the ISCN 2016 nomenclature.

### Copy Number Variation Sequencing (CNV-Seq)

Briefly, genomic DNAs were extracted using the Whole Blood DNA kit (BGI, Shenzhen, China) according to the manufacturer’s protocol. The DNA quality and concentration were assessed using a Qubit 2.0 fluorometer (Thermo Fisher Scientific, Waltham, MA, United States). Approximate 5 million sequencing reads per sample were mapped to the NCBI human reference genome (hg19/GRCh37) using the Burrows-Wheeler Aligner (BWA) tool and then allocated to 20 kb sequencing bins with 5 kb sliding to achieve higher resolution for CNV detection. The CNV-seq profiles of each chromosome were represented as log2 of the mean CNV of each sequencing bin along the length of the chromosome.

Sequence variants were annotated using population and literature databases including DECIPHER^1^, DGV^2^, 1000 Genomes Project^3^, OMIM^4^, ClinVar^5^, ClinGen^6^, and ISCA CNV^7^. Online software was used to analyze the structures of proteins, predict the conserved and functional domains and perform multiple sequence alignment. CNVs were classified into three categories (benign, uncertain clinical significance and pathogenic) according to the American College of Medical Genetics and Genomics (ACMG) standards and guidelines for the interpretation of CNVs.

### Follow-Up

The outcomes of fetuses with EIF were followed by telephone. Follow-up was conducted when the children were 3–6 months, 6–12 months, and 1–2 years of age. The results of ultrasonography and physical examinations after birth were recorded.

### Statistics

Identifiable personal information was removed from the data used in the analysis to protect individuals’ privacy. The data are presented as mean with median and n (% or ‰). Statistical significance was evaluated using the Student’s *t*-test, Chi-square test or Fisher’s exact test, and *p* < 0.05 was considered statistically significant.

## Results

### Characteristics of Patients

From January 2018 to April 2020, 3,377 invasive pregnancy diagnoses were performed in our center. Among them, 571 (571/3,377, 16.9%) fetuses with USMs (USMs) were enrolled in this study. EIFs, which were detected in 150 fetuses, comprised 4.44% (150/3,377) of the entire diagnosis population and 26.27% (150/571) of the fetuses with USMs. The mean ages of the pregnant women with EIF and USM groups were 29.72 and 29.67 years, respectively (the EIF group ranged from 21 to 44 years, median 29 years, *SD* = 4.91; the USMs group ranged from 18 to 44 years, median 29 years, *SD* = 4.67). Student’s t-test revealed no significant differences in the ages of these two groups (*p* = 0.912). As shown in Table 1, 82 (82/150, 54.67%) cases had only one EIF, and 68 (68/150, 45.33%) cases showed multiple EIFs by ultrasound. The locations of the EIFs were recorded; most of them were in left ventricle (113/150, 75.33%), some were in both ventricles (31/150, 20.67%), and others were found in right ventricle (6/150, 4.00%).

**TABLE 1:** Characteristics of EIFs in fetuses.

**Types**	**No. of patients**
Total	150
**Focus number**	
Single EIF	82 (54.67%)
Multi EIFs	68 (45.33%)
**Focus position**	
Left ventricle	113 (75.33%)
Right ventricle	6 (4.00%)
Both ventricles	31 (20.67%)

### Chromosomal Abnormalities in Fetuses With EIFs

The main chromosomal abnormalities detected through prenatal diagnosis were abnormal karyotypes and pCNVs (pathogenic CNVs and likely pathogenic CNVs). As shown in Table 2, among the 571 fetuses with USMs, a total of 44 (44/571, 7.71%) chromosomal abnormalities were identified; 28 (28/571, 4.90%) were detected by karyotype analysis, including 16 cases of trisomy 21 (16/28, 57.14% of karyotype abnormalities, one presented a Robertson translocation between chromosome 14 and 21), 5 cases of trisomy 18 (5/28, 17.86% of karyotype abnormalities), 6 cases of sex chromosome aneuploidies (6/28, 21.43% of karyotype abnormalities, of which 3 were mosaics), and one case with large fragment deletion in chromosome 18 (1/28, 3.57% of karyotype abnormalities). 16 (16/571, 2.80%) anomalies were pathogenic or likely pathogenic CNVs (pCNVs) which smaller than 5 Mb. In which, one case (1/16, 6.25% of pCNVs)detected microdeletion related to a X-linked ichthyosis, one case (1/16, 6.25% of pCNVs) was Y chromosome microdeletion related to spermatogenic failure, two were chromosome 22q11.2 microduplication syndromes (2/16, 12.50% of pCNVs), two cases detected chromosome 16p11.2 deletion syndromes (2/16, 12.50% of pCNVs), pCNVs in three cases were microdeletions on chromosome 15 (3/16, 18.75% of pCNVs), two pathological/likely pathological microdeletions were on chromosome 17 (2/16, 12.50% of pCNVs), two were microdeletions on chromosome 9 (2/16, 12.50% of pCNVs), and 3 pCNVs were found on chromosome2 10, and 20, respectively (Table 3 and Supplementary Table 1).

**TABLE 2:** Incidence of chromosomal abnormalities in fetuses with EIFs and USMs.

	**No. of patients**	**No. of patients with chromosomal abnormalities (% of No. of patients)**	***p***	**No. of patients with abnormal karyotype (% of No. of patients)**	***p***	**No. of patients with < 5Mb pCNVs (% of No. of patients)**	***p***
USMs	571	44 (7.71)		28 (4.90)		16 (2.80)	
EIFs	150	6 (4.00)	0.112^a^	2 (1.33)	0.051^a^	4 (2.67)	1.000^a^
Isolated EIFs	59	0		0		0	
With other USMs	91	6 (6.59)	0.082^b^	2 (2.20)	0.5200^b^	4 (4.40)	0.154^b^
Gestational weeks <28	135	3 (2.22)		2 (1.48)		1 (0.74)	
Gestational weeks ≥28	15	3 (20.00)	0.014^c^	0	1.000^c^	3 (20.00)	0.003^c^
Age <35	122	5 (4.10)		2 (1.64)		3 (2.46)	
Age ≥35	28	1 (3.57)	1.000^d^	0	1.000^d^	1 (3.57)	0.566^d^

*^*a*^Compared with USM group, by Chi-square (X^2^) test.*

*^*b*^Compared with isolated EIF group, by fisher’s exact test.*

*^*c*^Compared with gestational weeks <28, by fisher’s exact test.*

*^*d*^Compared with maternal age <35, by fisher’s exact test.*

**TABLE 3:** Chromosomal abnormalities and pregnancy outcomes of fetuses withEIFs.

**Case No.**	**Maternal age (years)**	**Ultrasound indications**	**CNV-seq results**		**Pathogenicity**	**Karyotype**	**Pregnancy outcome**
						
			**Segments of CNVs**	**Clinical phenotype for ultrasound findings**			
18S4620963	30	Echogenic intracardiac focus, echogenic bowel, cerebral ventriculomegaly	Trisomy 21	Down’s syndrome	Pathogenic	47,XY, + 21	Labor induction
18S4364136	23	Echogenic intracardiac focus, thicken of nuchal translucence	Trisomy 21	Down’s syndrome	Pathogenic	47,XY, + 21	Labor induction
19S2775503	27	Echogenic intracardiac focus, nasal bone dysplasia, echogenic bowel	del(17p11.2p12). seq[GRCh37/hg19] (14,989,438–16,852,433) (1.86 Mb)	–	Pathogenic	46,XX	Labor induction
20S2508263	41	Echogenic intracardiac focus, mild pyelectasis	del(15q11.2).seq[GRCh37/hg19] (22,646,193–23,514,853) (868.66 Kb)	Congenital heart disease	Likely pathogenic	46,XY	Full-term delivery, develop normal at 3 months
19S3641497	33	Echogenic intracardiac focus, permanent right umbilical vein	46,XN,dup(22q11.21).seq[GRCh37/hg19] (18,765,311–21,630,621) (2.87 Mb)	Congenital heart disease	Pathogenic	46,XY	Refused to disclose
18S3921249	28	Echogenic intracardiac focus, mild pyelectasis	del(9p22.3).seq[GRCh37/hg19] (14,263,199–15,390,161) (1.13 Mb)	Renal hypoplasia	Likely pathogenic	46,XX	Refused to disclose

For further investigation, we focused on the association of EIFs and chromosomal abnormalities. Compared to fetuses with USMs, 6 of the total 150 fetuses (6/150, 4.00%) with EIFs suffered from chromosomal abnormalities, but the difference was not significant (4.00 vs. 7.71%, *p* = 0.112). In detailed, the chromosomal abnormalities included 2 (2/150, 1.33%) karyotype abnormalities (both were trisomy 21) and 4 (4/150, 2.67%) pCNVs, similar to the USMs group (Table 2).

Subsequently, the fetuses with EIFs were divided into two groups based on existence of other USMs. Among the 91 fetuses who presented other USMs beside EIFs, chromosomal abnormalities were detected in 6 (6/91, 6.59%) fetuses, including 2 cases of trisomy 21 (2/91, 2.20%) and 4 cases of pCNVs (4/91, 4.40%); while no abnormal karyotype or pCNVs were found in 59 fetuses showed isolated EIFs.

Finally, we focused on the relationship between fetal EIFs and gestational stage/mother age. When scanned by ultrasound, 135 EIF cases were found and referred to prenatal diagnosis at the second trimester (gestational stage ranged from 18 to 26 weeks); the EIFs in the remaining 15 fetuses were detected until the late second trimester, and the invasive diagnosis was conducted over 28 weeks of gestation (from 28 to 31 weeks). The incidence of chromosomal abnormalities was dramatically increased in group over 28 weeks of gestation (2.22% in less than 28 weeks of gestation vs. 20.00% in over 28 weeks of gestation, *p* = 0.014). This discrepancy could be attributed to the number of pCNVs, for example, only 1 out of 135 (0.74%) pregnancies before 28 weeks of gestation were found pCNVs, and this incidence increased to 20.00% (3 in 15) in the pregnancies over 28 weeks (*p* = 0.003). On the other hand, there was no difference in the observed incidence of chromosomal abnormalities in fetuses with EIFs when the maternal ages were below or above 35 years (4.10 vs. 3.57%, *p* = 1.000, Table 2).

### Pregnancy Outcomes

Six chromosomal abnormalities in the group of fetuses with EIFs and the outcomes of these pregnancies were shown in Table 3. Three cases terminated pregnancy, 2 refused to disclose the pregnancy outcome, and 1 fetus obtained the same likely pCNV from the mother and was followed up to 3 months after birth. In the latter case, physical examination was normal, but ultrasound examination could not be conducted.

In addition, 144 fetuses without pCNVs were followed up from birth to 2 years of age. Of these, 127 were born full term, 5 were born prematurely at 33–36 W of gestation, 3 mothers chose labor induction, and 9 patients refused to disclose their pregnancy outcomes. As shown in Table 4, 25 children underwent re-examination by ultrasound after birth. Among them, 9 cases (6.25%, 9/144) of congenital heart disease were found, in which 1 patent foramen ovale self-cured at 9 months, 1 case had a valve defect and accepted cardiac surgery at 1 year old, 2 displayed persistent EIFs without obvious heart defects, and 14 showed no heart defects (the oldest was over 2 years of age at the last examination and developed normally). In addition, although no re-examination after birth, 22 pregnant women accepted ultrasound screening several weeks after invasive prenatal diagnosis, EIFs were still detected in 6 (6/22, 27.27%) of these fetuses, whereas disappeared in 16 (16/22, 72.73%) fetuses. The incidence of congenital heart disease after birth was not different in fetuses with a single EIF and those with more than one EIF (*p* = 1.000). In the group that received ultrasound before birth, the ratio of EIF disappearance on re-examination was similar in fetuses who showed only one EIF and those with multiple EIFs (*p* = 0.136).

**TABLE 4:** Re-examination results by ultrasound.

	**No. of patients**	**Single EIF**	**Multi EIFs**	p*
Ultrasound after birth	25	10	15	
Congenital heart disease	9	4	5	1.000
Patent foramen oval	4	2	2	
Ventricular septal defect	3	1	2	
Valve defect	1	0	1	
Right aortic arch	1	1	0	
Echogenic intracardiac focus	2	0	2	
Normal	14	6	8	
Ultrasound before birth	22	12	10	
With EIF	6	3	3	0.136
Without EIF	16	9	7	

*Compared between fetuses with single EIF and multi EIFs.

## Discussion

The current study investigated fetuses who presented EIFs in prenatal diagnosis. From year 2018 to 2020, among 3,377 cases of prenatal diagnosis in our center, the incidence of EIFs identified by sonographic screening was 4.44% (150 in 3,377); and the most common cardiac lesions were in the left ventricle (75.33%), in accordance to the previous literatures (How et al., 1994; Bromley et al., 1995, 1998; Wax et al., 2000; Nyberg et al., 2001; Wang et al., 2018). In our data, EIFs, comprised 26.27% of USMs (150 in 571), were one of the most prevalent USMs in fetuses. Moreover, in clinical consulting, it is confusing when EIFs present without other information available from serum screening or without association with a definite structural anomaly by ultrasound. Thus, this investigation focused on fetuses who presented EIFs while patients do not have other definite or high-risk indications for chromosomal abnormalities.

Here, we analyzed the risk of fetal chromosomal abnormalities by both karyotyping and CNV-seq. Among a total of 571 pregnancies that displayed USMs, beside 28 fetuses with karyotype abnormalities (28/571, 4.90%), 16 (16/571, 2.80%) extra pathogenic CNVs were identified. The pCNVs founded here can result in a wide range of syndromes including X-linked ichthyosis, spermatogenic failure, 22q11.2 microduplication syndromes, 16p11.2 deletion syndrome, etc. Notably, we did not detect pCNVs cause serious heart defects in fetuses showed EIFs, such as microdeletions on 22q11.2 for DiGeorge syndrome. This may due to our exclusion of samples who presented structure defects under ultrasound screening. To be noted, besides the USM indications, including EIFs, other phenotypes can be detect by CNV-seq. For example, the detection of two 16p11.2 microdeletion syndromes came from both fetuses displayed external left superior cavity, which has no relation with typical epilepsies or intellectual disability of 16p11.2 microdeletion syndromes; and in fetuses showed thickened nuchal translucence, a wide spectrum of pCNVs involved from spermatogenesis to mental development was found; in fetuses showed EIFs and other USMs simultaneously, two pCNVs had no correlation with their ultrasound results were detected. Thus, the application of CNVs detection can expand the detection area of syndromes in prenatal diagnosis, and the symptoms of underlying syndromes may not only restrict in ultrasound findings. Moreover, our data is in agreement with previous reports based on chromosomal microarray analyses, which demonstrated that fetuses with abnormal ultrasound findings included 2.8–3.5% pathogenic CNVs that were not detectable by karyotyping (Wapner et al., 2012; Society for Maternal-Fetal Medicine et al., 2016; Lostchuck et al., 2019). Therefore, although aneuploidy in EIF fetuses has been extensively studied, subchromosomal abnormalities still need to be evaluated.

In fetuses with EIFs, the incidence of chromosomal abnormalities was 4.00% which is not different from the whole USM group (vs. 7.71%, *p* = 0.125), and is also similar to other soft markers that had been proposed previously (Nyberg et al., 2001). In EIF group, among 59 fetuses showed isolated EIFs, no chromosomal abnormality was found. Our data support the idea that EIFs alone is not indicative for an increased risk of chromosomal abnormalities (Bromley et al., 1998; Manning et al., 1998; Lamont et al., 2004; Bradley et al., 2005; Van Den Hof et al., 2005).

It is generally accepted that nearly half of EIFs observed in the second trimester may resolve with advancing gestation, although the genetic characteristics of the remaining EIF-positive fetuses are still not clear (Hurd and Nelson, 2009; Su et al., 2011). We analyzed the results of 15 invasive diagnosed cases conducted at late gestational stages in which echogenic foci persisted through the late second to third trimester. No aneuploidy was detected at gestational stages over 28 weeks, and this was not different from the earlier gestational age group. Interestingly, 20% incidence (3 in 15 cases) of pCNVs in the late gestational age group was significantly higher than that was found in earlier gestational age group (0.74%, *p* = 0.003). This result suggests that EIFs, especially appeared simultaneously with other USMs that persist to late gestational ages could indicate higher risk of pathogenic CNVs which may be missed by conventional karyotyping. In addition, because of its shorter reporting cycle compared to karyotyping, CNV-seq has more advantages for patients at late gestational stages. This finding emphasizes the efficiency of CNV-seq in a field in which conventional karyotyping was previously considered standard.

Maternal age is generally assumed as an important factor that associated with the aneuploidy rate in fetuses with EIFs (Bromley et al., 1998; Goncalves et al., 2006). However, when we analyzed the data on the basis of maternal age (younger vs. older age), no correlation of age was found. This may be due to the relatively low incidence of aneuploidy in our study, which is attributed to the exclusion of some high-risk samples by serum screening, NIPT, or fetal structural anomalies, etc. The incidence of pCNVs was also similar in old and young patients, in according with the idea that CNVs can occur in any pregnancy independent of maternal ages (Chau et al., 2019).

In our clinical management, the parents of fetuses who have been detected of carrying pCNVs were suggested to take the verification of CNVs. Meanwhile, the ultrasound results throughout pregnancy were considered comprehensively together with pCNVs during clinical consulting. Moreover, we have conducted telephone calls to follow up with the outcomes of fetuses with pCNVs. It was regrettable that the pregnancy terminations were not conducted in our hospital; for this reason, autopsies to verify the dysplasia could not be performed. In one case of full-term birth, though the infant possessed a likely pathogenic CNV that was inherited from the mother, the infant behaved normally and showed normal development according to gross physical examination. To be noted, cardiac ultrasound was not performed because the infant was only 3 months old at the time of follow-up, furthermore, the development of intelligence and language could not be assessed.

Previously, EIF was considered as a normal developmental variant that was not associated with congenital heart disease (Simpson et al., 1996; Wolman et al., 2000; Wax et al., 2003). However, in clinical consulting, cases with EIFs were found to be associated with heart defects, causing confusion and possibly leading to poor prognosis. A conflicting conclusion was reached that the prevalence of cardiac defects in fetuses with EIFs was twice than that in the general newborn population (Goncalves et al., 2006). Furthermore, some benign defects, such as ventricular septal defect and patent foramen ovale maybe self-cure prior to 3 years of age (Jortveit et al., 2016; Cho et al., 2017) and might not be observed at the time of re-examination, could cause an artificial decrease of the heart defect incidence. Here, we followed the outcomes of fetuses and found that among 25 neonates who underwent cardiac ultrasound prior to 6 months of age, 9 (36.00%) presented abnormalities, mostly with patent foramen ovale (44.44%) and ventricular septal defects (33.33%). This result was relatively higher than expected and may be that the population underwent re-examination after birth including cases with persisted EIFs throughout the pregnancy. It is reported that 8–75% of EIFs disappear as gestation progresses (Arda et al., 2007; Lorente et al., 2017); a similar percentage was found in our study, in which 72.73% of EIFs could not be detected in re-emanations after invasive prenatal diagnosis. Thus, we calculated that the percentage of heart defects in the total population in this study was 6.25%, higher than the 0.8% observed in the general population (Prefumo et al., 2003). This was consistent with previous reports, suggests that EIFs in fetuses may be associated with the presence of heart defects (Lorente et al., 2017; Guo et al., 2018).

In conclusion, by applying high-resolution sequencing simultaneously with conventional karyotyping, additional chromosomal anomalies were detected in our study. The still-controversial association between EIFs and chromosomal abnormalities was reevaluated using data on CNVs. Isolated EIFs appear not to indicate increased risk of chromosomal abnormalities. Interestingly, although most EIFs disappeared with advanced stages of gestation, EIFs detected with other USMs that persisted in late trimester may indicate the presence of pathogenic CNVs in the fetus. Thus, at late gestational ages, CNVs detection has its advantages over karyotyping. By analyzing the follow up data, more congenital heart disease was found after birth in fetuses with EIFs than in fetuses with normal conception and no ultrasound abnormality, a comprehensive sonographic examination throughout the entire pregnancy and after birth could be recommended. Our investigation provides detailed information from the perspective of genetic disorders and prognosis after birth of fetuses with EIFs for clinical consulting.

## Data Availability Statement

According to the national legislation/guidelines, specifically the Administrative Regulations of the People’s Republic of China on Human Genetic Resources (http://www.gov.cn/zhengce/content/2019-06/10/content_5398829.htm, http://eng lish.www.gov.cn/policies/latest_releases/2019/06/10/content_281476708945462.htm), no additional raw data are available at this time. The data presented in the study are temporarily deposited in the BGI data delivery system (http://cdts.genomics.org.cn/). The accession code 20210310F12FHQHSSJ0877B and the password ZhaoYanF12345! should be included in the application, or you can email to the corresponding authors for details.

## Ethics Statement

The studies involving human participants were reviewed and approved by the Ethics Committee of First Affiliated Hospital of the University of Science and Technology of China (USTC). The patients/participants provided their written informed consent to participate in this study.

## Author Contributions

YS and JW conceived and designed the study. YS, JX, and XJ wrote the manuscript. HL performed the ultrasound scan. JG and XJ contributed to manuscript revision. JW, LW, and GH provided clinical information of patients and conducted invasive diagnosis. YS, JX, WL, YH, and YP performed the experiments and genetic detections. YS and FY completed the follow ups. All authors contributed to the article and approved the submitted version.

## Conflict of Interest

The authors declare that the research was conducted in the absence of any commercial or financial relationships that could be construed as a potential conflict of interest.

## Footnotes

1 https://decipher.sanger.ac.uk/

2 http://dgv.tcag.ca/

3 http://www.internationalgenome.org/

4 http://omim.org/

5 http://www.ncbi.nlm.nih.gov/clinvar

6 https://www.clinicalgenome.org/

7 https://www.iscaconsortium.org
