# Supplementary material for: Application of Copy Number Variation Detection to Fetal Diagnosis of Echogenic Intracardiac Focus During Pregnancy
Source: Front Genet. 2021 Mar 26;12:626044. doi: 10.3389/fgene.2021.626044 (PMC8047624; doi:10.3389/fgene.2021.626044)
Supplement: Supplementary file 1 [file Table_1.DOCX]

**Supplementary Table 1** pCNVs in fetuses with USMs (not include EIFs).

| **Case No.** | **Ultrasound indications** | **CNV-seq results** | | **Pathogenicity** |
| --- | --- | --- | --- | --- |
| 19S3641467 | echogenic bowel, enhanced renal cortex echo | 46,XN,del(17q12).(34,429,429-36,219,068)×1 | 1.79Mb | Pathogenic |
| 19S5159032 | external left superior cavity | 46,XN,del(16p11.2).(29,506,514-30,169,599)×1 | 663.09Kb | Pathogenic |
| 19S2775459 | external left superior cavity | 46,XN,del(16p11.2).(29,578,974-30,194,495)×1 | 615.52Kb | Pathogenic |
| 19S5158831 | external left superior cavity | 46,XN,del(20p12.1p12.2).(9,565,036-12,260,249)×1 | 2.70Mb | Pathogenic |
| 19S3641555 | nasal bone dysplasia | 46,XN,del(Xp22.31).(6,420,555-8,229,370)×(0~1) | 1.81Mb | Pathogenic |
| 19S5159075 | nasal bone dysplasia | 46,XN,dup(22q11.21).(18,759,074-21,764,954)×3 | 3.01Mb | Pathogenic |
| 19S2775602 | thickened nuchal translucence | 46,XY,del(Yq11.223q11.23).(24,026,787-26,227,153)×0 | 2.20Mb | Pathogenic |
| 19S2775179 | thickened nuchal translucence | 46,XN,dup(2q13).(111,179,721-113,139,673)×3 | 1.96Mb | Likely Pathogenic |
| 19S2775496 | thickened nuchal translucence | 46,XN,del(10p15.3).(60,466-2,219,833)×1 | 2.16Mb | Pathogenic |
| 19S5159080 | thickened nuchal translucence | 46,XN,del(9p21.2).(26,993,630-27,179,353)×1 | 185.72Kb | Likely Pathogenic |
| 19S3641531 | thickened nuchal translucence | 46,XY,del(15q25.2q25.3).(81,838,907-86,796,598)×1 | 4.96Mb | Pathogenic |
| 19S5158789 | thickened nuchal translucence | 46,XN,del(15q11.2).(22,618,483-23,299,051)×1 | 680.57Kb | Likely Pathogenic |
